# Host-Specific Enzyme-Substrate Interactions in SPM-1 Metallo-β-Lactamase Are Modulated by Second Sphere Residues

**DOI:** 10.1371/journal.ppat.1003817

**Published:** 2014-01-02

**Authors:** Lisandro J. González, Diego M. Moreno, Robert A. Bonomo, Alejandro J. Vila

**Affiliations:** 1 Instituto de Biología Molecular y Celular de Rosario (IBR, CONICET-UNR) and Area Biofísica, Facultad de Ciencias Bioquímicas y Farmacéuticas, Universidad Nacional de Rosario, Rosario, Argentina; 2 Instituto de Química Rosario (IQUIR, CONICET-UNR), Facultad de Ciencias Bioquímicas y Farmacéuticas, Universidad Nacional de Rosario, Rosario, Argentina; 3 Research Service, Louis Stokes Cleveland Department of Veterans Affairs Medical Center and Departments of Medicine, Pharmacology, Molecular Biology and Microbiology, Case Western Reserve University, School of Medicine, Cleveland, Ohio, United States of America; Massachusetts General Hospital, Harvard Medical School, United States of America

## Abstract

*Pseudomonas aeruginosa* is one of the most virulent and resistant non-fermenting Gram-negative pathogens in the clinic. Unfortunately, *P. aeruginosa* has acquired genes encoding metallo-β-lactamases (MβLs), enzymes able to hydrolyze most β-lactam antibiotics. SPM-1 is an MβL produced only by *P. aeruginosa*, while other MβLs are found in different bacteria. Despite similar active sites, the resistance profile of MβLs towards β-lactams changes from one enzyme to the other. SPM-1 is unique among pathogen-associated MβLs in that it contains “atypical” second sphere residues (S84, G121). Codon randomization on these positions and further selection of resistance-conferring mutants was performed. MICs, periplasmic enzymatic activity, Zn(II) requirements, and protein stability was assessed. Our results indicated that identity of second sphere residues modulates the substrate preferences and the resistance profile of SPM-1 expressed in *P. aeruginosa*. The second sphere residues found in wild type SPM-1 give rise to a substrate selectivity that is observed only in the periplasmic environment. These residues also allow SPM-1 to confer resistance in *P. aeruginosa* under Zn(II)-limiting conditions, such as those expected under infection. By optimizing the catalytic efficiency towards β-lactam antibiotics, the enzyme stability and the Zn(II) binding features, molecular evolution meets the specific needs of a pathogenic bacterial host by means of substitutions outside the active site.

## Author Summary

The presence of Zn(II)-containing metallo-β-lactamases (MβLs) that confer resistance to all penicillins, cephalosporins and carbapenems in *Pseudomonas aeruginosa* adds significantly to the threat of this pathogen in our health care system. SPM-1 is an MβLs widely distributed in South America and only found in *P. aeruginosa*. In common with all MβLs, the active site residues are highly conserved. In this work we asked the following question: how would substrate specificity evolve in SPM-1 if the active site residues are highly uniform and do not permit substitutions. To this end, we explored the role of two amino acids (S84 and G121) that are outside the active site (second sphere) and are unique in the SPM-1 β-lactamase. We discovered that replacing these amino acids impacts resistance to cephalosporins and carbapenems and that this resistance profile depends on the enzymatic behavior and the availability of Zn(II) in the environment. This work demonstrates how protein evolution by means of subtle substitutions outside the active site meets the specific needs of a pathogenic bacterial host.

## Introduction

β-lactam antibiotics (penicillins, cephalosporins, monobactams and carbapenems) are the most dependable and frequently employed chemotherapeutic agents for eradicating bacterial infections [1]. Their safety and efficacy as antimicrobial agents derives from their ability to selectively inhibit cell wall biosynthesis, provoking bacterial cell wall lysis [2]. The newest types of β-lactam antibiotics (e.g. carbapenems) and expanded-spectrum cephalosporins (e.g. cefepime), evade most common mechanisms of resistance against these compounds [3]. These compounds are currently used as “last resort” drugs for treating multi-resistant gram-negative pathogens [1], [3].

The major mechanism of resistance against β-lactam antibiotics is the production of bacterial β-lactamases which catalyze cleavage of the antibiotic β-lactam ring rendering an inactive derivative [2]. β-lactamases fall into four classes (A–D). Classes A, C and D are serine-β-lactamases (SβLs) which employ an active-site serine to catalyze antibiotic hydrolysis, while metallo-β-lactamases (MβLs), or class B β-lactamases, are metallo-enzymes requiring one or two zinc ions for their activity [4].

MβLs gained importance in the 1990s as the principal mechanism of resistance against carbapenems (imipenem, meropenem) [5], [6], [7]. MβLs degrade all classes of β-lactams except monobactams and, unlike most SβLs, these enzymes are not susceptible to therapeutic β-lactamase inhibitors. This fact, together with the facile dissemination of MβL genes among different clinical pathogens, relegates them as a serious clinical threat [5], [7]. Indeed, outbreaks of pathogens producing NDM-1, IMPs, VIMs or SPM-1 MβLs are increasingly common worldwide [8].

**Figure 1:**
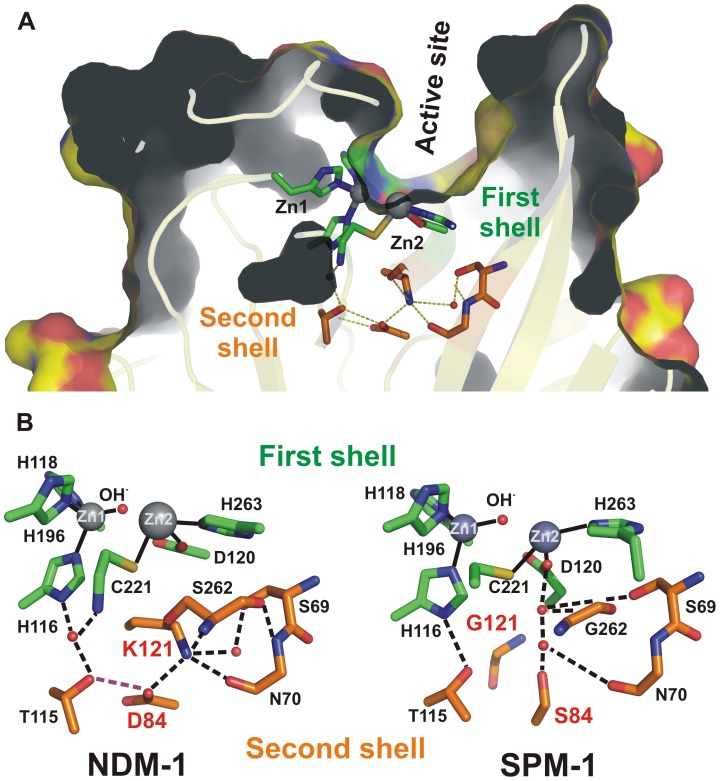
(A) Location of first shell (green) and second shell (orange) residues in the structure of SPM-1. **(B)** Schematic representation of the active sites of the B1 MβLs NDM-1 and SPM-1 emphasizing the differences in the hydrogen bond networks involving metal ligands (H116, H118, H196, D120, C221 and H263, in green) and second sphere residues (115, 84, 121, 69, 70 and 262, in orange) due to residues 84 (D or S) and 121 (K or G).

Atomic structures reveal that clinically relevant MβLs (subclass B1) possess similar active sites: indeed, residues binding the essential Zn(II) ions (first sphere residues) are strictly conserved (Figure 1A) [6], [7]. Despite being “broad-spectrum” enzymes, MβLs exhibit quite different substrate profiles, which cannot be correlated to different active site structures [9]. Many structural and mechanistic studies have focused on the analysis of active site residues and the role of active-site flanking loops to account for substrate recognition of MβLs [6], [9], [10]. However, the mechanism by which different B1 enzymes are tailored to hydrolyze some antibiotics better than others is not known. The fundamental question remains: *how does protein evolution occur among MβLs that are found exclusively and adapted to a particular host*? This problem represents a central issue in linking molecular features to organismal behavior. In the clinic this notion may contribute to therapeutic failure.

*Pseudomonas aeruginosa* is one of the most clinically important non-fermenting Gram-negative pathogens, being well known for its ability to acquire genes encoding resistance determinants, such as the acquired MβLs [11], [12]. In addition, *P. aeruginosa* harbors a host of virulence factors. Of particular relevance, SPM-1 is an MβL produced only by *P. aeruginosa*, while other MβLs have been found in many different bacterial hosts [12], [13], [14], [15], [16], [17], [18]. At the present time, the *bla*_SPM-1_ gene is associated with a single clone (SP/ST 277) of *P. aeruginosa*. This clone emerged relatively recently in South America. This unique dissemination suggests either: 1) the *bla*_SPM_ gene came from another organism and has expanded in SP/ST 277 because of a fitness advantage; or that 2) this genetic determinant may have been optimized to meet the need of its native host. SPM-1 in *P. aeruginosa* is therefore a unique system to analyze the role of host-specific constraints in molecular evolution.

The structure of SPM-1 has revealed unique features among pathogen-associated MβLs [19]. Spencer and coworkers have shown that clinically relevant B1 enzymes share a hydrogen bonding network spanning below the active site base, generally known as second sphere residues (Figure 1) [19]. This network is disrupted in SPM-1 due to the presence of two atypical second sphere residues: S84 and G121, which replace the conserved D84/R121 couple (Figure 1B) [9].

Here we examine the role of these positions (located outside the enzyme active site “in the second sphere”) and their impact on antibiotic resistance in the native bacterial host, *P. aeruginosa*. We report that this unique combination of residues is able to provide resistance to anti-pseudomonal β-lactams such as latest generation cephalosporins and carbapenems [11], while sacrificing the catalytic efficiency against other β-lactams. Our findings reveal that second sphere residues are able to modulate the substrate specificity of MβLs according to the requirements of the bacterial host In addition, we show that these second sphere residues optimize the zinc binding affinity of SPM-1 in the bacterial periplasm, providing *P. aeruginosa* antibiotic resistance under zinc-limiting conditions, such as those prevalent during bacterial infection [20], [21].

## Results

### Mimicking the natural host of SPM-1

**Figure 2:**
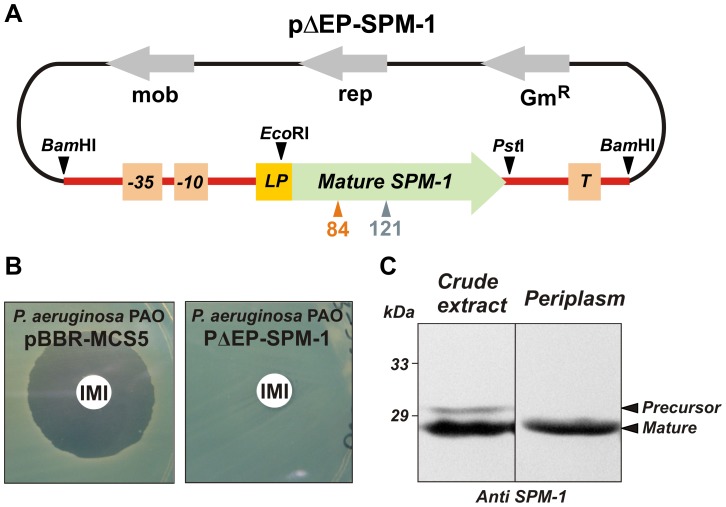
(A) Plasmid pΔEP-SPM-1, showing the transcriptional unit (UT *bla*_SPM-1_ in red) harboring gene *bla*_SPM-1_. **(B)** Imipenem antibiograms of *P. aeruginosa* PAO containing plasmids pBBR-MCS5 and pΔEP-SPM-1. (C) Immune detection of SPM-1 in a crude extract and periplasmic fraction of *P. aeruginosa* PAO pΔEP-SPM-1.

*E.coli* is usually employed as a model bacterial host to compare the ability of the different MβLs to confer resistance, even for enzymes which are not found in Enterobacteriaceae [9]. We designed a system aimed to reproduce the native conditions of expression of the *bla*_SPM-1_ gene. The complete *bla*_SPM-1_ transcriptional unit from the clinical strain *P. aeruginosa* 48-1997A [15] (*i.e.*, including the natural promoter, the leader peptide for periplasmic location, the mature protein and the transcriptional terminator) was amplified and subcloned into the broad-spectrum vector pBBR1-MCS5 [22], replicative in *P. aeruginosa* PAO (Figure 2A). *P. aeruginosa* PAO cells transformed with this vector (pΔEP-SPM-1) were able to express SPM-1, export and process it properly to the periplasmic space. Western blot analysis showed two SPM-1 forms of 30.6 and 27.5 kDa in whole cell extracts, corresponding to the precursor and mature species respectively [13]. Instead, the periplasmic fraction contained only the mature form of the enzyme (Figure 2B,C). Accordingly, the transformed cells were resistant to imipenem.

### Codon randomization and selection of resistance-conferring mutants

In order to assess the “flexibility” of positions 84 and 121 in accommodating residues different from the native ones (S84 and G121), codons 84 and 121 were individually randomized in *bla*_SPM-1_ by overlap-extension PCR [23], [24]. The amplifications were targeted to the mature *bla*_SPM-1_ coding sequence, and then subcloned into the screening vector, so as to avoid undesired mutations in promoter and terminator sequences. In addition, codons 84 and 121 were randomized together looking for possible synergistic effects between these positions.

Single-codon random libraries gave rise to 10^3^–10^4^ transformants, while the double-codon mutant library elicited >3×10^4^ transformants. According to *Poisson* distribution, the libraries obtained have a probability of harboring a mutant *bla*_SPM-1_ gene with a specific codon at position 84 or 121 (or a specific combination of codons) >99% [23]. Sequences of ten randomly selected mutants from each library indicated no obvious bias.

**Figure 3:**
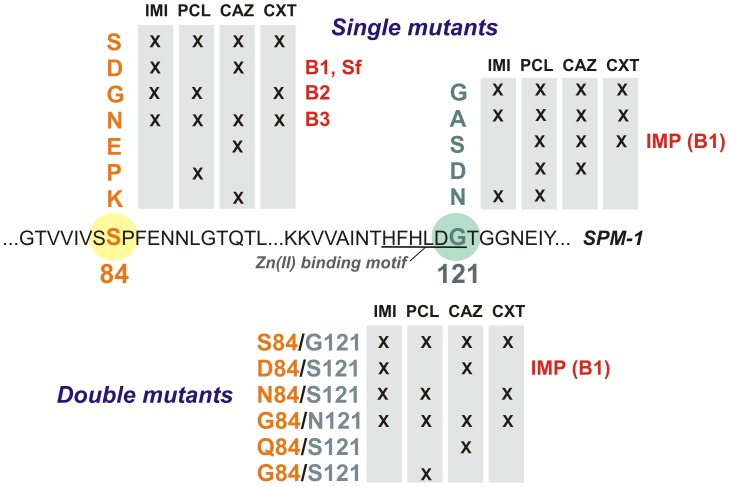
Residues present at positions 84 and 121 in selected mutants for each antibiotic selection and library in *P. aeruginosa* PAO. IMI = imipenem, PCL = piperacillin, CAZ = ceftazidime, CXT = cefoxitin.

Active mutants were selected by examining the ability of the different libraries to confer resistance toward different types of β-lactam antibiotics in *P. aeruginosa* PAO (Figure 3). Paper discs embedded with different antibiotics were applied onto LB-Gm agar plates with *P. aeruginosa* PAO transformed with the randomized libraries. We employed a penicillin (piperacillin), a third-generation cephalosporin (ceftazidime), a cephamycin (cefoxitin), and a carbapenem (imipenem).

Twenty bacterial clones exhibiting resistance (*i.e.*, located within the halos) were isolated for each library and for each tested antibiotic (a total of 240 clones). Plasmids were extracted and *bla*_SPM-1_ was sequenced in each clone. In total, 16 different variants (wild type SPM-1, 10 single mutants and 5 double mutants) were isolated. As expected, wild type clones (with residues S84 and G121) were selected in all cases. Mutants G121A, S84N and S84N/G121S were also selected against all tested antibiotics (Figure 3). On the other hand, some substitutions were isolated depending on the screening antibiotic, implying that positions 84 and 121 modulate the substrate profile of the enzyme. Surprisingly, none of the selected mutants carried mutations G121R or G121H (prevalent in B1 and B3 enzymes, respectively). Substitutions at position 84, instead, displayed typical residues from B1 (S84D), B2 (S84G) and B3 enzymes (S84N), among others [9]. The S84D/G121S combination is present in the B1 enzyme IMP-1, closely related to SPM-1 [13]. We then analyzed the resistance profile of the libraries.

### Second sphere mutations affect substrate-dependent resistance in *P. aeruginosa*

**Figure 4:**
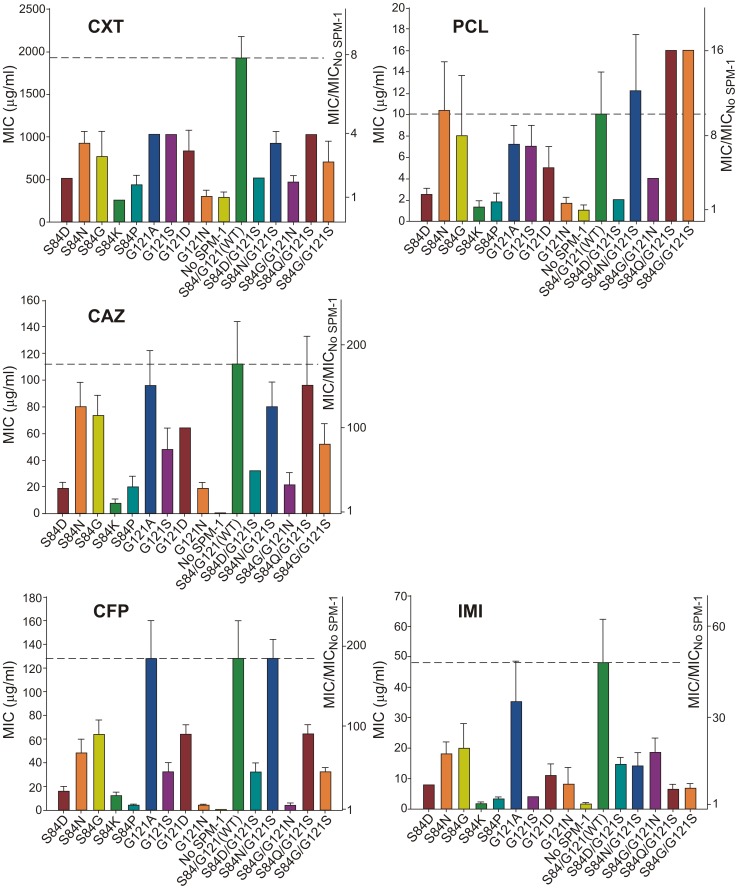
Minimum inhibitory concentration (MIC) of imipenem (IMI), piperacillin (PCL), ceftazidime (CAZ), cefoxitin (CXT) and cefepime (CFP) for selected strains of *P. aeruginosa* (producing the different SPM-1 mutants at positions 84 and 121). No SPM-1 = *P. aeruginosa* containing the vector pBBR-MCS5. Values come from 5 independent determinations.

MIC values for *P. aeruginosa* cells expressing each of the selected SPM-1 mutants were determined against different antibiotics. Cefepime (an antipseudomonal cephalosporin) was added to the initial set of antibiotics. Expression of SPM-1 markedly increased resistance towards antipseudomonas drugs such as ceftazidime and cefepime (200–250 times), while for cefoxitin (an antibiotic to which *P. aeruginosa* PAO is naturally resistant), the increase in MIC was only 7-fold (Figure 4).

In general, single-codon variants S84G, S84N (naturally present in B2 and B3 enzymes) and G121A (the most conservative substitution in this position) display the highest MIC values after the wild type (WT) enzyme (MIC values equal or up to 2-dilutions lower compared to WT SPM-1). In fact, together with S84N/G121S, these mutants were the most ubiquitous in the antibiotic selection experiments. Synergistic effects between residues 84 and 121 are apparent when comparing double mutants *vs.* single mutants. For example, while S84G and G121S mutations were detrimental for resistance against piperacillin (MIC values approximately half a dilution lower than for WT), the combination of both mutations generated an enzyme conferring higher levels of resistance than the wild type (MIC value of 16 µg/ml for S84G/G121S vs. 10 µg/ml for WT SPM-1) (Figure 4). Surprisingly, the S84D/G121S combination, naturally occurring in IMP enzymes, was not among the most resistant mutants for any of the antibiotics assayed (MIC values 2–3 dilutions lower compared to WT SPM-1).

Figure 4 summarizes our data showing that mutations had different impact in the bacterial resistance profile depending on the antibiotic (selection criteria). Therefore, second sphere positions 84 and 121 are able to shape the resistance profile, and possibly the substrate specificity. In general, mutants conferred lower levels of resistance than wild type SPM-1 (within a range of 5 dilutions in MIC values). Surprisingly, piperacillin is an exception, since four mutants outperform the wild type variant (S84N, S84N/G121S, S84Q/G121S and S84G/G121S in up to half a dilution in MIC values). In the case of cefoxitin, the range of MIC values spanned by the different variants is smaller than for the rest of the tested antibiotics (3 dilutions vs. 4 or 5 dilutions). The impact of mutations on MIC values is more informative for the case of the antipseudomonas compounds ceftazidime and cefepime, where several single and double mutants provide levels of resistance comparable to the WT enzyme, with MIC values increasing by two orders of magnitude. For imipenem, G121A is the only mutant giving rise to a large MIC value (35 µg/ml vs. 48 µg/ml for WT SPM-1).

### Enzymatic activity *in periplasma* parallels the resistance profile and reveals that SPM-1 is tailored to improve resistance against drugs with anti-pseudomonal activity

Enzymatic studies *in vitro* of MβLs have been useful to uncover structural and mechanistic aspects of these enzymes. However, these data rarely correlate with the *in vivo* behavior [9]. We attempted to correlate the MIC values with the hydrolytic profile of the different SPM-1 mutants assayed in periplasmic extracts of *P. aeruginosa*, *i.e.*, in an environment closer to *in vivo* conditions. The β-lactamase activity of SPM-1 mutants was assayed in periplasmic extracts of *P. aeruginosa* PAO (*in periplasma*) and normalized relative to the amount of enzyme present in the periplasm (quantitated from Western blot gels). Given that SPM-1 is an efficient cephalosporinase *in vitro*, we focused on these substrates. We employed three substrates with antipseudomonal activity already used in the MIC experiments: ceftazidime, cefepime and the carbapenem drug imipenem, together with two first-generation cephalosporins devoid of antipseudomonal activity (cephalexin and cephalothin).

**Figure 5:**
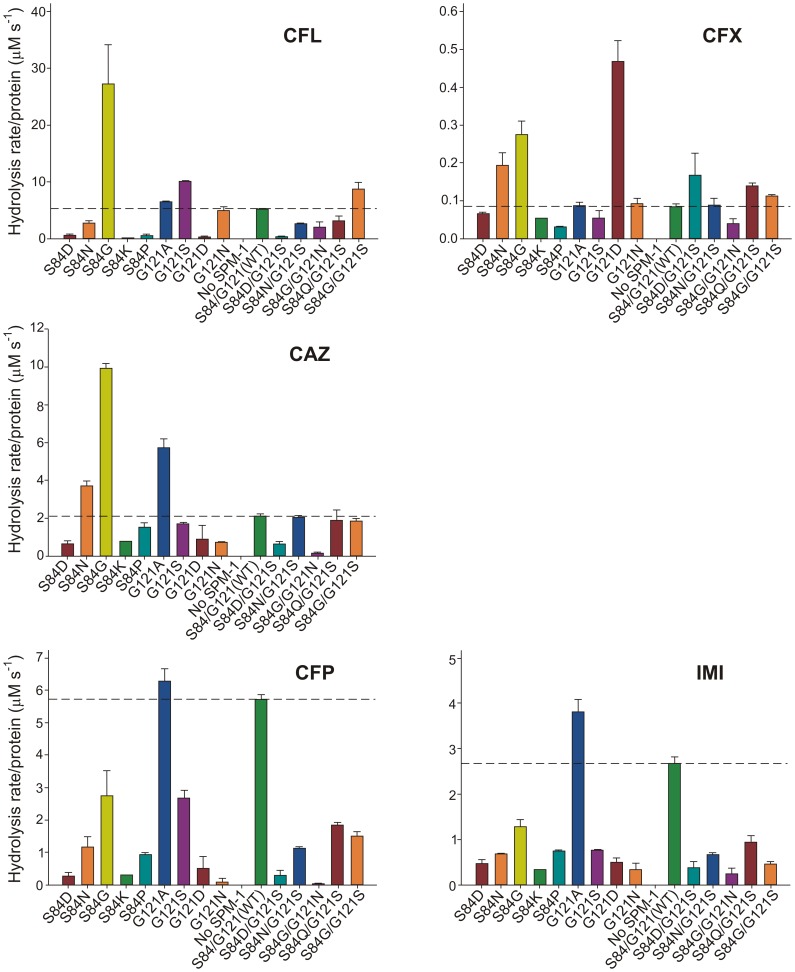
β-lactamase activity of SPM-1 mutants in *P. aeruginosa* periplasmic extracts against cephalotin (CFL), cephalexin (CFX), ceftazidime (CAZ), cefepime (CFP) and imipenem (IMI). Rates of hydrolysis are normalized to the amount of periplasmic protein amounts (Materials and Methods). Reactions were accomplished in Tris 10 mM, MgCl_2_ 30 mM pH 7.3 and 800 µM antibiotic. Error bars come from activity measurements from five independent periplasmic fractionations.

Hydrolysis rates *in periplasma* show a very good correlation with MIC values in the case of cefepime and imipenem (Figure 5). For these two substrates, only mutant G121A was competitive with the performance of wild type SPM-1. Instead, in the case of ceftazidime, cephalotin and cephalexin, the hydrolytic performance of the wild type enzyme was surpassed by several mutants. Mutant S84G (present in B2 enzymes), and to a lesser extent S84N (present in B3 enzymes) were the variants eliciting the best performance for first-generation cephalosporins. We conclude that the second coordination sphere modulates the substrate specificity in SPM-1 so that this enzyme is adapted to better hydrolyze the latest antipseudomonal antibiotics (cefepime and imipenem) while the catalytic performance against first-generation drugs is far from being optimized. In all cases, the activity of endogenous AmpC was negligible (as revealed by the lack of activity in the “No SPM-1” control strains).

**Figure 6:**
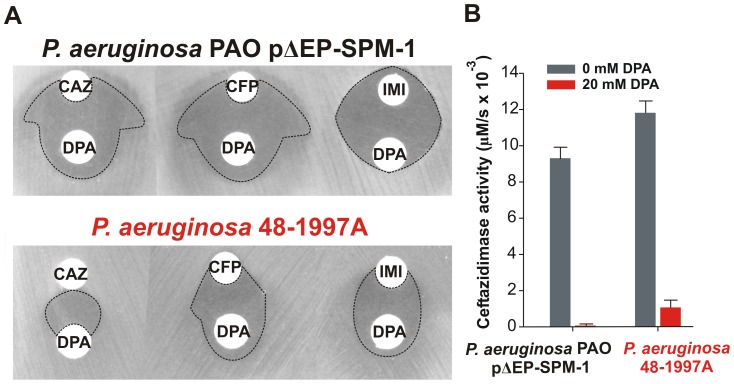
(A) Antibiograms of *P. aeruginosa* PAO pΔEP-SPM-1 and *P. aeruginosa* 48-1997A against disks embedded with 30 µg ceftazidime (CAZ), 30 µg cefepime (CFP) and 10 µg imipenem (IMI), alone or faced to 1.5 mg dipicolinic acid (DPA) containing disks. Halos of inhibition are remarked with dotted lines. (B) Ceftazidime hydrolysis rates (300 µM) of crude extracts of *P. aeruginosa* PAO pΔEP-SPM-1 and *P. aeruginosa* 48-1997A, with and without 20 mM DPA. Reaction medium was 10 mM Tris, 30 mM MgCl_2_ at pH 7.3.

Ceftazidime shows a different profile: albeit being an antipseudomonal drug, can be better hydrolyzed by several mutants than by native SPM-1. However, *P. aeruginosa* has developed different resistance mechanisms against ceftazidime which do not affect cefepime and imipenem (hyperproduction of endogenous AmpC, deregulation of efflux pumps or acquisition of ESBLs) [11]. We therefore evaluated the role of SPM-1 in the resistance of the *P. aeruginosa* clinical strain against these three antibiotics. Disks embedded with ceftazidime, cefepime and imipenem were paired with disks containing dipicolinic acid (DPA, an inhibitor of SPM-1), on an agar plate inoculated with the clinical strain *P. aeruginosa* 48-1997A (*including its native bla_SPM-1_ gene*) and the control *P. aeruginosa* PAO expressing SPM-1 [25]. While halos of inhibition were similar for all antibiotics in the control strain, ceftazidime exhibited a reduced halo in the clinical strain (Figure 6). When DPA was added to whole cell extracts of the model strain, no residual activity was monitored. Instead, residual ceftazidime hydrolysis was present after addition of DPA to extracts from the clinical strain (Figure 6). We conclude that resistance against ceftazidime in the clinical strain is not exclusively due to expression of SPM-1, and therefore this drug has not elicited a significant evolutionary pressure on this enzyme (or a higher activity against this antibiotic was not necessary a part of the substrate spectrum in order to be acquired by *P. aeruginosa*). In fact, there is evidence of SPM-1 producing isolates of *P. aeruginosa* that express AmpC (probably due to an hyperproduction phenotype) and OXA-52 enzymes, supporting our hypothesis [26].

We postulate that SPM-1 in the bacterial host has been exposed to the evolutionary pressure of the administration of newest antibiotics such as cefepime and imipenem, thus adapting to better hydrolyze these compounds by changes in the second coordination sphere. At this point, it is intriguing that mutant G121A, providing high levels of resistance and hydrolysis rates in the periplasm, has not been selected during natural evolution.

### Limiting Zn(II) conditions have exerted evolutionary pressure in selecting SPM-1 as the optimal variant in *P. aeruginosa*

**Figure 7:**
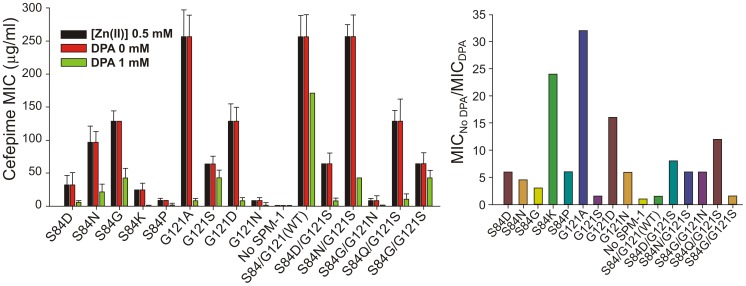
MIC values against cefepime of *P. aeruginosa* PAO containing SPM-1 mutants at different concentrations of the metal chelator dipicolinic acid (DPA).

MβLs are exported to the bacterial periplasm as unfolded polypeptides [27]. Therefore, in the apo (non-metallated) form, metal site assembly (giving rise to the active variants) takes place in the periplasmic space [27]. We have recently shown that Zn(II) availability is limited in this compartment, and that MβLs with reduced Zn(II) binding capabilities are unable to confer resistance [28]. We determined the MIC values of *P. aeruginosa* cells with different SPM-1 mutants in media containing excess or limiting concentrations of Zn(II) against cefepime. MIC values were unaffected by Zn(II) supplementation in all cases. However, under metal deprivation conditions (by adding the chelating agent DPA), strikingly distinct effects were observed for the different SPM-1 variants (figure 7).

Mutant G121A, exhibiting a high specific activity *in periplasma* (Figure 5), was the most sensitive variant to metal deprivation (MIC values are 64-fold lower in 1 mM DPA) followed by variants S84K and G121D. Wild type and mutant G121S, on the other extreme, were almost unaffected by these conditions (MICs values diminished in only one-dilution in 1 mM DPA). The bacterial growth was also unaffected by these conditions as assayed by MIC values for the strain without the SPM-1 expression system. The lack of effect of excess Zn(II) in bacterial resistance likely reflects the action of the CzcABC pump in *P. aeruginosa* which, by extrusion of excess Zn(II) into the extracellular medium, keeps constant the levels of periplasmic Zn(II).

Thus, the second coordination sphere exquisitely tunes the zinc binding ability of SPM-1 so that the enzyme has been evolved to provide resistance at metal limiting concentrations. As a result, the atypical S84/G121 combination allows SPM-1 to confer antibiotic resistance under these conditions.

### Protein stability determines the Zn(II) binding affinity

*P. aeruginosa* PAO periplasmic extracts revealed similar levels of periplasmic SPM-1 variants, with the exception of S84P and S84K mutants, which were undetectable. We analyzed the thermal stability of the mutants *in periplasma*, by studying the temperature dependence of (1) periplasmic β-lactamase activity or (2) SPM-1 solubility for each mutant. Hydrolysis rates were evaluated at 30°C after incubation at different temperatures. Instead, protein solubility was analyzed by Western blot quantitation of the levels of soluble SPM-1 variants after incubation at different temperatures. A plot correlating the hydrolytic activities (or solubilities) with the incubation temperature revealed in all cases a well-behaved sigmoidal behavior, that can be fit to obtain apparent *Tm* (*Tm_app_*) values for each variant (Figure S1 and Table 1). *Tm_app_* values determined from both strategies display an astonishingly good correlation.

**Table 1:** Values of *Tm_app_* for different SPM-1 mutants and their apo derivatives, measured in periplasmic extracts of *P. aeruginosa* PAO by β-lactam hydrolysis rates and protein solubility.

	*Tm_app_* (°C)			
SPM-1 variant	Holo-derivative		Apo-derivative	
	Activity	Solubility	Activity	Solubility
S84D	57.4±1.8	62.3±2.0	46.0±2.8	49.9±2.8
S84G	69.7±1.7	68.5±1.9	53.1±2.1	50.2±2.2
S84/G121 (WT)	71.9±2.0	73.1±1.6	46.9±2.0	55.5±2.1
G121A	61.0±1.6	62.5±1.5	-*	-*
G121S	77.1±1.0	78.1±1.5	44.2±2.1	48.0±2.3
G121D	55.0±2.7	53.2±2.0	-*	-*
S84D/G121S	55.3±2.3	56.4±2.5	42.5±2.0	53.1±2.1
S84K	58.0±2.0	nd	nd	nd
S84P	60.4±3.0	nd	nd	nd
S84N	64.6±0.4	nd	nd	nd
G121N	63.5±1.7	nd	nd	nd
S84N/G121S	64.0±0.7	nd	nd	nd
S84G/G121S	71.3±0.6	nd	nd	nd
S84Q/G121S	65.0±1.9	nd	nd	nd
S84G/G121N	66.8±2.8	nd	nd	nd

^nd^Not determined.

^*^The apo derivatives of these mutants precipitated during metal removal.

The four most stable periplasmic SPM-1 variants were G121S>S84/G121 (WT)≈S84G/G121S≥S84G, while the combination S84D/G121S (naturally found in IMP-1) was the least stable mutant together with G121D and, to a lesser extent, G121A. At this point we selected some representative mutants for further characterization (S84D, S84G, G121A, G121S, G121D, G121N, S84D/G121S and wild type).

Similar experiments were performed with the apo-derivatives of periplasmic SPM-1 variants, which were obtained by dialyzing the periplasmic fractions against EDTA and DPA metal-chelators, excess NaCl and finally metal-free reaction buffer. *Tm_app_* values of the apo variants were estimated as before by β-lactam activity (in this case supplementing reaction media with 2 µM Zn(II)) and protein solubility (Table 1).

Apo-derivatives exhibited a narrower range of *Tm_app_* values, suggesting that the main differences observed in the stability of holo-derivatives are due to stabilization upon metal binding. Mutant G121S showed the largest metal-induced stabilization (∼30°C difference in *Tm_app_* between holo and apo derivatives). Mutant S84D/G121S, on the other extreme, was marginally stabilized by metal binding. Mutants G121A and G121D precipitated during Zn(II) removal. Mutants showing larger differences in stabilities between the apo and holo form are expected to be those displaying large Zn(II) binding affinities. In agreement with this hypothesis, the variants exhibiting the highest metal-induced stabilization (wild type SPM-1, S84G, G121S and S84G/G121S) were the least sensitive to metal deprivation (Figure 7).

**Figure 8:**
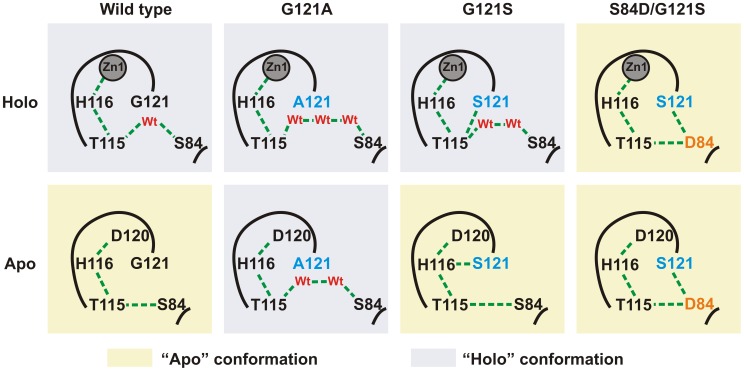
Simplified scheme of the H-bridge interaction network present in the different variants of SPM-1 under study. Holo and apo-like conformations are highlighted in blue and yellow, respectively.

We explored the structural effects of these mutations by molecular dynamics (MD) simulations in wild type SPM-1 and three selected variants: G121A, G121S and S84D/G121S [19]. After 5 ns of MD simulations, a water molecule from the bulk solvent penetrates the active site of holo-wild type SPM-1, occupying the vacant position between T115 and S84 (Figure S2), and reconstructing the hydrogen bond network present in all B1 enzymes [19]. In the apo forms, Zn1 ligands become mobile, mostly due to alterations in second sphere residues. The largest changes were observed in the cavity between residues T115 and S84. In WT SPM-1, the second sphere adopted two different conformations: (a) “holo-like”, in which the cavity was able to accommodate water molecules connecting residues T115 and S84, and (b) “apo-like”, in which water molecules were excluded from this network, and T115 and S84 show a direct interaction (Figures 8 and S3). The holo and apo variants of G121S (the mutant showing the highest metal-induced stabilization) closely resemble the structure of the holo and apo forms of WT SPM-1. Instead, in the case of the S84D/G121S, the second sphere residues are locked into an “apo-like” conformation, disfavoring metal binding. In the case of G121A, the Ala121 side chain avoids contraction of the cavity, locking the second sphere into the “holo-like” form. The low stability of apo G121A suggests that this conformation is not viable in the apo form.

## Discussion

SPM-1 from *P. aeruginosa* is unique among pathogen-associated MβLs in presenting the singular S84/G121 combination as second sphere residues, instead of the conserved D84/R121 couple [13], [19]. Here we report a thorough study of the impact of mutations in these positions in the antibiotic resistance, specific enzymatic activity, metal binding features and protein stability.

A major novelty in our approach is the extensive use of the native host, *P. aeruginosa*. This approach allowed us: (1) to perform a medium-throughput screening of activities and stabilities of a series of mutants with a high degree of reproducibility, (2) to correlate enzymatic activities reproducing the native conditions (i.e., within the cell) during bacterial growth, which parallel the resistance profile and (3) to identify additional environmental factors which may not stem out from *in vitro* studies or by using *E.coli* as a model bacterial host [9]. In fact, MIC values of imipenem elicited by MβLs in *E. coli* are markedly lower to those determined in their natural hosts [9], [29]. In the particular case of MβLs, these enzymes are active only when the Zn(II) availability in the periplasm allows proper metal uptake, therefore being much more dependent on the bacterial host than serine lactamases [27], [28].

Expression of WT SPM-1 in *P. aeruginosa* selectively raises the MIC values against ceftazidime, cefepime and imipenem. These MIC values correlate with the *in periplasma* specific activities, in particular against cefepime and imipenem. The wild type variant, together with G121A, shows the highest specific activities against these two antibiotics. Instead, many single and double mutants in positions 84 and 121 outperformed wild type SPM-1 versus several antibiotics to which *P. aeruginosa* is intrinsically resistant. This remarkable substrate selectivity control by second sphere residues shows that the atypical S84/G121 combination present in SPM-1 has been fixed to provide resistance to anti-pseudomonal drugs, while sacrificing the catalytic efficiency against other antibiotics.

Analysis of the resistance profile against cefepime by controlling the Zn(II) availability in the external medium reveals that G121A (the only variant able to compete with wild type SPM-1 in terms of specific activity) is extremely sensitive to metal deprivation. The fact that G121A is not a natural variant of SPM-1 despite the high resistance observed in metal-rich media suggests that evolutionary pressure has been exerted to select MβL variants capable of providing resistance in low Zn(II) environments. Native SPM-1, instead, is able to confer resistance under conditions of Zn(II) deficiency. Indeed, during infection, the immune system produces large amounts of calprotectin, a host-defense protein that prevents bacterial colonization by chelating Mn(II) and Zn(II) [20], [21]. Thus, optimization of the zinc binding capabilities is a crucial evolutionary trait for MβLs in their natural environment. This finding, together with a recent report highlighting the need of proper assembly of a dinuclear site in the active site of MβLs in the periplasm [28], highlights the need to address the periplasmic bacterial mechanism of Zn(II) homeostasis and its role in antibiotic resistance, which have been largely overlooked.

The role of second sphere residues in catalysis is an emerging issue in enzymology [30]. A hydrogen bond network connecting metal binding residues below the active site is meant to preserve the electrostatics and modulate the active site features. Directed evolution experiments on the B1 enzyme BcII enzyme revealed that mutations responsible of enhancing the lactamase activity were located in this hydrogen bond network [31], [32]. As analyzed in detail by Spencer [19] and Oelshlaeger [33], [34] in structural, modeling and mutagenesis studies, this network spans metal ligands His116, Asp120 and Cys221, and the second sphere residues 115, 84, 121, 69, 70 and 262. The D84/R121 combination is the most commonly found in B1 enzymes [35]. Molecular dynamics simulations showed that water molecules can enter into the second sphere hydrogen bond network in SPM-1. These calculations also support how changes in the second sphere can modulate the Zn(II) binding affinity, ultimately impacting in the resistance profile in limiting metal environments.

Most acquired MβLs, such as enzymes from the IMP, VIM and NDM families present many allelic variants, in contrast to SPM-1 [36]. This difference could be due to the fact that SPM-1, as we demonstrate here, is optimized to meet specific *Pseudomonas* requirements, in contrast to the other MβL genes, present in many different genera of bacteria.

In a broader perspective, our approach allowed us to investigate how resistance determinants adapt to specific host requirements, linking fine details of the structural and biophysical features of the enzymes with bacterial fitness. More studies using this approach are required to account for the versatility and adaptability of MβLs to overcome the challenge imposed by new antibiotics.

## Materials and Methods

### Ethics statement

Rabbits were housed and treated according to the policies of the Canadian Council on Animal Care guidelines on: antibody production (http://www.ccac.ca/Documents/Standards/Guidelines/Antibody_production.pdf). All efforts were made to minimize suffering and the procedures were approved by the Bioethics Commission for the Management and Use of Laboratory Animals inside the Science and Technical Committee of the University of Rosario, under resolution number 490/2012 (PICT-2008-N°0405).

### Bacterial strains

*Escherichia coli* DH5α (Gibco- BRL, Gaithersburg, MD, U.S.A.) was used for construction of pΔEP-SPM-1 plasmid. *Pseudomonas aeruginosa* 48-1997A, originally identified in Brazil, was provided by M. Castanheira and M. Toleman [13], and used as the source of *bla*_SPM-1_. Laboratory strain *P. aeruginosa* PAO was used for transformation of mutant libraries, microbiological and biochemical studies. All strains were grown aerobically at 37°C in lysogeny broth (LB) medium supplemented with antibiotics when necessary.

### Recombinant DNA methodology

Molecular biology procedures were done according to Sambrook *et al.* Transcriptional unit of *bla*_SPM-1_ was PCR-amplified from a genomic preparation of *P. aeruginosa* 48-1997A using primers SPM-1-fw and SPM-1-rv (Table 2), both containing a *Bam*HI restriction site, and subcloned into pBBR1-MCS5 plasmid [22]. The product was digested with *Xho*I and *Sma*I enzymes (Promega) to eliminate restriction sites *Eco*RI and *Pst*I from the MCS of the plasmid. Extremes were made blunt by treatment with Klenow fragment (Promega) and then ligated with T4 DNA ligase (Promega). Restriction sites *Eco*RI and *Pst*I were introduced at the each edge of SPM-1 coding sequence by mutagenesis using primers *Eco*RI-fw, *Eco*RI-rv, *Pst*I-fw and *Pst*I-rv (Table 2). The resultant plasmid, pΔEP-SPM-1, was introduced into *P. aeruginosa* PAO by electroporation as described [37].

**Table 2:** Primers used in this work.

1	SPM-1-fw	AGCTGGATCCCGCGACAAGGCCCAACTCAC
2	SPM-1-rv	AGCTGGATCCCACCACCGAGGGCTCTTCAC
3	EcoRI-fw	CGGAGATCGGAATGAATTCACCTAAATCGAGAG
4	EcoRI-rv	CTCTCGATTTAGGTGAATTCATTCCGATCTCCG
5	PstI-fw	CTCACATCCCCAACCCTGCAGTTAACGCTTTCAAAC
6	PstI-rv	AGTCAGTCCTCGAGGGTTGGGGATGTGAGACTAC
7	S84X-fw	GACCGTTGTCATTGTCNNNTCGCCGTTTGAAAATCTG
8	S84X-rv	GATTTTCAAACGGCGANNNGACAATGACAACGGTCCC
9	G121X-fw	GCACTTTCATTTGGACNNNACGGGTGGAAATGAAATTTAC
10	G121X-rv	TTCATTTCCACCCGTNNNGTCCAAATGAAAGTGC

All constructs and amplifications were verified by sequencing at the University of Maine (Orono, USA).

### Codon randomization and selection of resistant clones

Codons corresponding to positions 84 and 121 (BBL numbering [35]) of SPM-1 were randomized individually by Overlap Extension PCR, as previously described [23], [24]. Mutagenic primers were designed so as to contain random trinucleotides at the desired positions (S84X-fw, S84X-rv, G121X-fw y G121X-rv, Table 2), using pΔEP-SPM-1 as the template [23], [24]. The products were subcloned into pΔEP-SPM-1 through *Eco*RI and *Pst*I restriction sites (thus avoiding unwanted mutations in promoter or terminator during PCR reactions), and the ligation mixtures electroporated in *P. aeruginosa* PAO. Electrocompentents from each mutant library (S84X and G121X) were spread in LB-agar plaques containing 30 µg/ml gentamicin, then collected and stored at −80°C. Library of double mutants S84X/G121X was constructed by submitting a plasmid preparation from S84X library to codon randomization of position 121, in the same way as before.

Selection of mutants capable of conferring some degree of resistance towards β-lactam antibiotics was done as follows. LB-agar plaques were inoculated with a bacterial culture (O.D. 0.1) of each mutant library, and disks embedded with 10 µg imipenem, 30 µg ceftazidime, 1000 µg cefoxitin, or 10 µg piperacillin placed on top of the agar. Mutant clones growing in the area of the antibiotic gradients were picked and the sequence of *bla*_SPM-1_ further determined [23].

### Microbiological assays

Production and/or resistance levels of SPM-1 in *P. aeruginosa* PAO pΔEP-SPM-1 or *P. aeruginosa* 48-1997A were assayed by pairing disks embedded with 1.5 mg dipicolinic acid (DPA) with disks containing 10 µg imipenem, 30 µg ceftazidime or 30 µg cefepime, onto LB-agar plaques inoculated with the corresponding bacterial culture (O.D. 0.1) [25], [38]. Minimal inhibitory concentrations (MICs) were determined on plaque by the dilution method [38].

### Cellular fractionation and protein level determinations

*P. aeruginosa* PAO crude extracts were obtained through sonication of cells washed in Tris 10 mM, MgCl_2_ 30 mM pH 7.3 followed by centrifugation at 4°C. Periplasmic preparations of *P. aeruginosa* PAO were obtained by shock with chloroform as previously described [39]. Contamination of periplasmic extracts with cytoplasmatic proteins was discarded by Western-blot with antibodies against cytoplasmatic DnaK [27].

Levels of periplasmic wild type SPM-1 and mutants were determined by Western-blot of periplasmic extracts with polyclonal antibodies against SPM-1 (obtained after inoculating a rabbit with a mixture of recombinant SPM-1 and Freund's adjuvant) and immunoglobulin G-alkaline phosphatase conjugate. Protein band intensities were quantified with the Gel-Pro Analyzer 4.0 software (Exon-Intron, Inc.) and normalized to a bacterial periplasmic protein arbitrarily chosen.

### β-lactamase activity

Initial rates of hydrolysis were measured in a JASCO V550 spectrophotometer at 30°C in 300 µl of reaction media containing 300 µM of substrate and 10 µl of *P. aeruginosa* PAO periplasmic or crude extract in 10 mM Tris, 30 mM MgCl_2_ at pH 7.3. For comparison, hydrolytic activities of periplasmic extracts were made relative to the amount of SPM-1 or mutant present in the extract, estimated by Western-blot anti-SPM-1 of the extracts normalized as before.

In order to study the contribution of SPM-1 in whole β-lactam activity, crude extracts (normalized in total protein concentration by Bradford assay [40]) were incubated during 20 minutes at room temperature with and without addition of 25 mM DPA, and initial rates measured and compared.

### Thermal denaturation of periplasmic extracts

Aliquots from each periplasmic extract of *P. aeruginosa* PAO were incubated for 5 minutes at various temperatures in the range 30–90°C, and then placed on ice for (a) determining initial rates of hydrolysis against ceftazidime, or (b) determining the levels of soluble SPM-1 or mutants (as before by Western-blot anti-SPM-1 of normalized extracts) after centrifugation for 10 min at 10,000 rpm and 4°C. Activity curves or soluble protein fraction as a function of temperature was adjusted to the sigmoid curve f = y0+a/(1+exp(−(x−x0)/b)) in Sigma Plot 9.0 program, with x0 the apparent melting temperature.

### Extraction of Zn(II) from periplasmic extracts

In order to generate apo-derivatives of periplasmic SPM-1 and mutants, periplasmic fractions of *P. aeruginosa* PAO were dialyzed in duplicate against 500 mM EDTA, 500 mM DPA, 50 mM Tris pH 8, then 2M NaCl, 50 mM Tris at pH 8, and finally 10 mM Tris, pH 7.3 30 mM MgCl_2_. The solutions were previously treated with chelating ion exchange resin (Chelex 100, Sigma-Aldrich) and dialysis times were of 6 hours.

### Molecular dynamic simulations

All simulations were performed in AMBER [41] starting from the crystal structure of SPM-1 determined with resolution of 1.9 Å (PDB code 2FHX) [19]. As crystallization of SPM-1 was achieved with a vacant Zn2 site, the metal site structure of SPM-1 was reconstructed by aligning it to the geometry of the Zn2 site of the homologous enzyme *B. cereus* BcII (PDB code 1BC2) [42]. In this way, a starting structure with a complete active site was obtained. Each simulation was performed using monomeric wild type SPM-1, or mutant proteins G121S, G121A, S84D/G121S modified *in silico*. Furthermore, three crystallographic azide molecules were replaced by water molecules (Wt1, Wt2 and Wt3) in the cavities present at the base of SPM-1 active site.

The systems were immersed in a box of water molecules TIP3P [43] and were simulated using periodic boundary conditions and Ewald sums for treating long-range electrostatic interactions [44]. The SHAKE algorithm was applied to all hydrogen-containing bonds [45]. This allowed us to use a time step of 2 fs for integration of Newton equations. Parm99 and TIP3P force fields implemented in AMBER were used to describe the protein and water, respectively [41]. The force field of the active site (Zn,-OH, Asp, Cys and His) was taken from the literature [46]. The temperature and pressure were controlled by the Berendsen thermostat and barostat respectively, as implemented in AMBER [41]. Cut-off values used for the van der Waals interactions were 10 Å. The systems were first minimized to optimize possible structural crashes and then slowly heated from 0 to 300 K under constant volume conditions, using a time step of 0.1 fs. Finally, a short simulation was conducted at a constant temperature of 300 K and under constant pressure of 1 bar, using a time step of 0.1 fs, to allow the systems reach a suitable density. These balanced structures were the starting points for the 10 ns of molecular dynamics simulations.

## Supporting Information

Figure S1 Thermal denaturation curves for wild type SPM-1, G121A, G121D, G121N, S84D, S84G and S84N/G121S mutants in periplasmic fractions. In red, curves obtained after treating periplasmic extracts with metal chelators. Below, SPM-1 Western-blots of the soluble fractions of the periplasmic extracts pre-incubated at different temperatures.
(TIF)

Figure S2 10 ns MD simulations of di-Zn(II) SPM-1. The cartoon indicates the labeling of the water molecules in the active site. After 5 ns of simulation, Wt4937 diffuses from the bulk solvent into the protein interior, reconstructing the conserved hydrogen bond network among second sphere residues.
(TIF)

Figure S3 Structures of holo and apo derivatives of wild type (WT), G121S, G121A and S84D/G121S SPM-1 after 10 ns of simulation. Metal-ligand bonds are shown in solid lines and second-shell interactions in dashed lines. On the right, evolution of H-O distances of possible H bridges between residues 115 and 84. For simplicity, Zn2 and Zn2-ligands C221 and H263 are omitted in most representations.
(TIF)
