# Supplementary figures and images for: Host-Specific Enzyme-Substrate Interactions in SPM-1 Metallo-β-Lactamase Are Modulated by Second Sphere Residues
Source: PLoS Pathog. 2014 Jan 2;10(1):e1003817. doi: 10.1371/journal.ppat.1003817 (PMC3879351; doi:10.1371/journal.ppat.1003817)

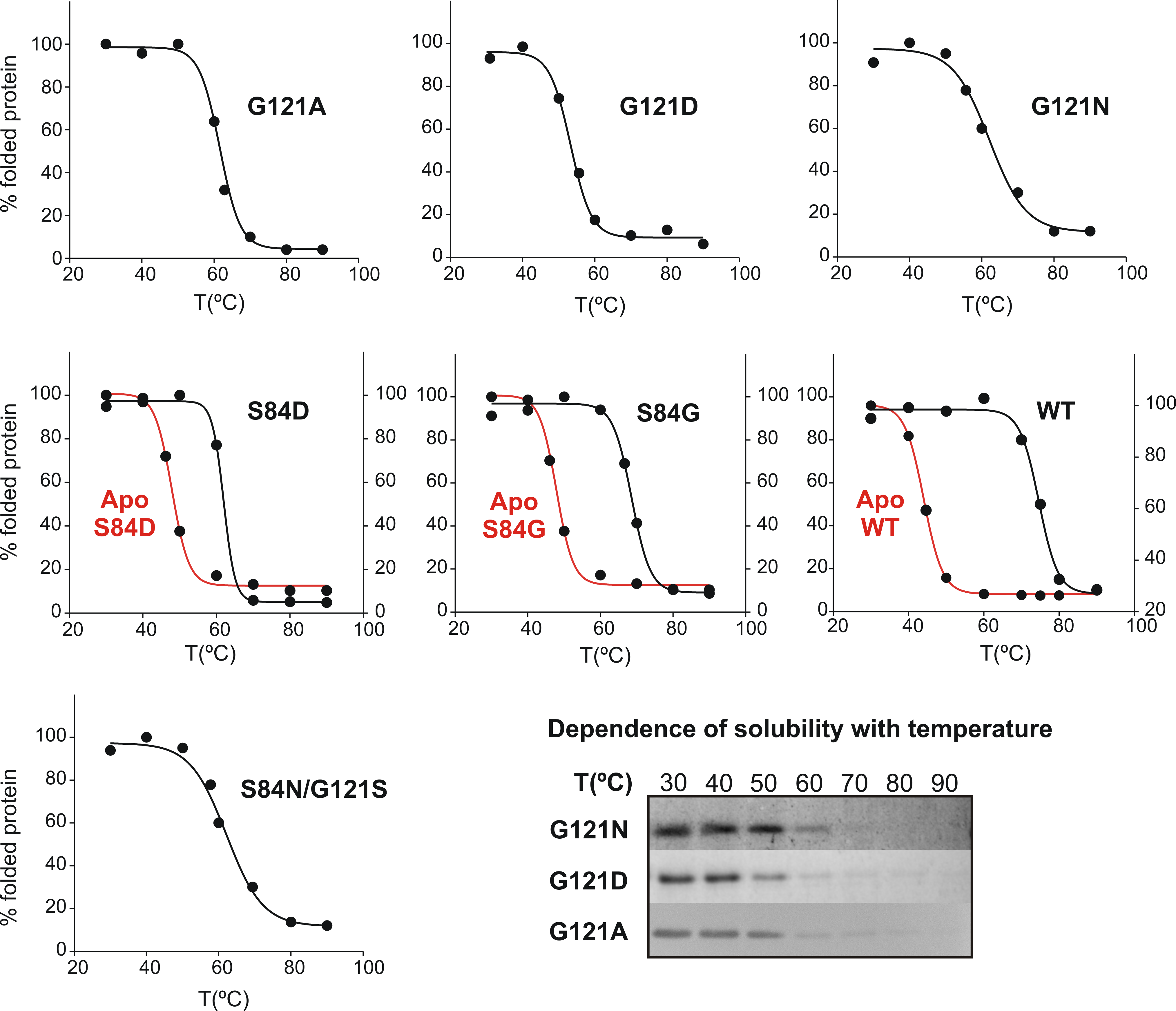

Supplement: Figure S1 — Thermal denaturation curves for wild type SPM-1, G121A, G121D, G121N, S84D, S84G and S84N/G121S mutants in periplasmic fractions. In red, curves obtained after treating periplasmic extracts with metal chelators. Below, SPM-1 Western-blots of the soluble fractions of the periplasmic extracts pre-incubated at different temperatures. (TIF) [file ppat.1003817.s001.tif]

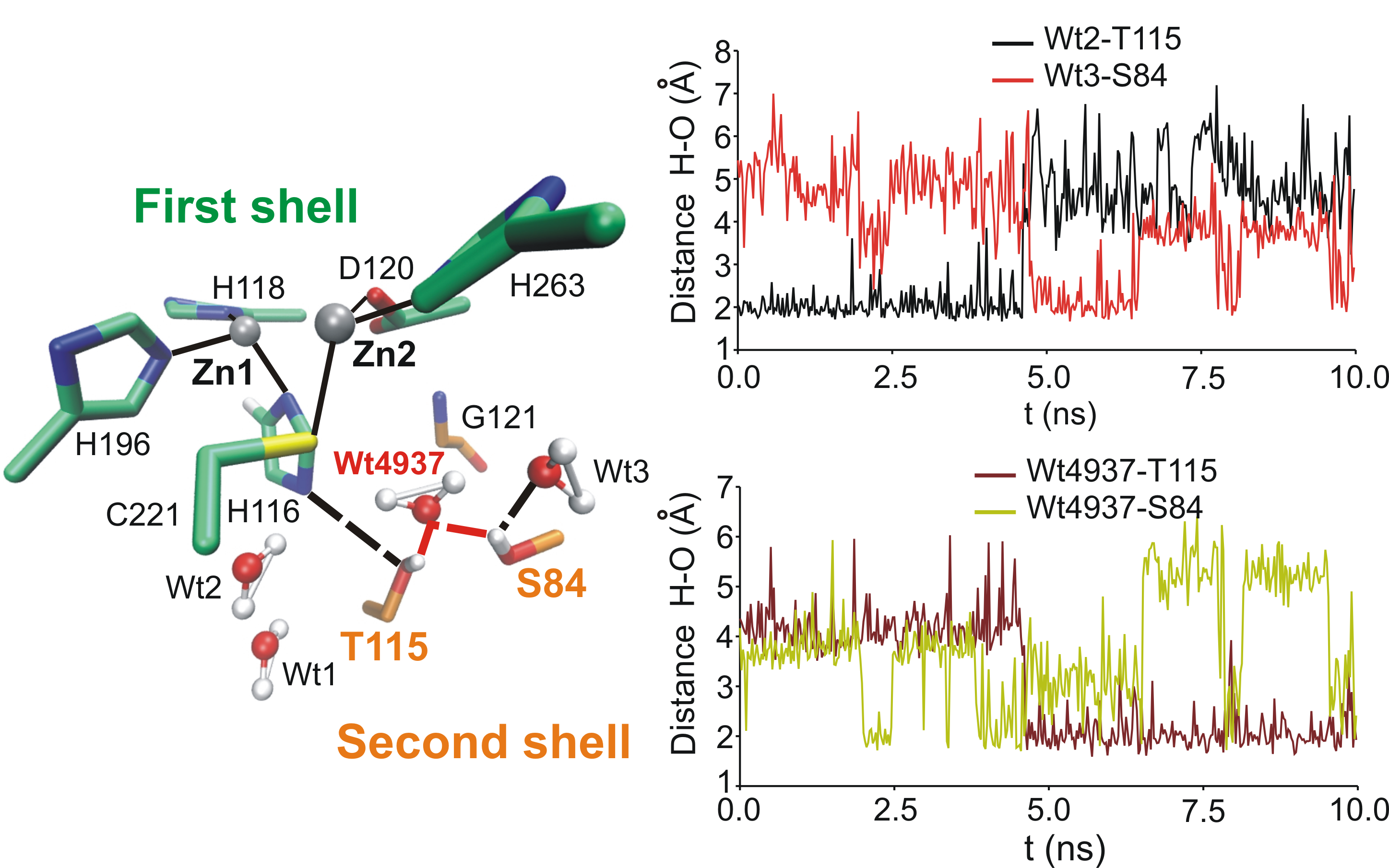

Supplement: Figure S2 — 10 ns MD simulations of di-Zn(II) SPM-1. The cartoon indicates the labeling of the water molecules in the active site. After 5 ns of simulation, Wt4937 diffuses from the bulk solvent into the protein interior, reconstructing the conserved hydrogen bond network among second sphere residues. (TIF) [file ppat.1003817.s002.tif]

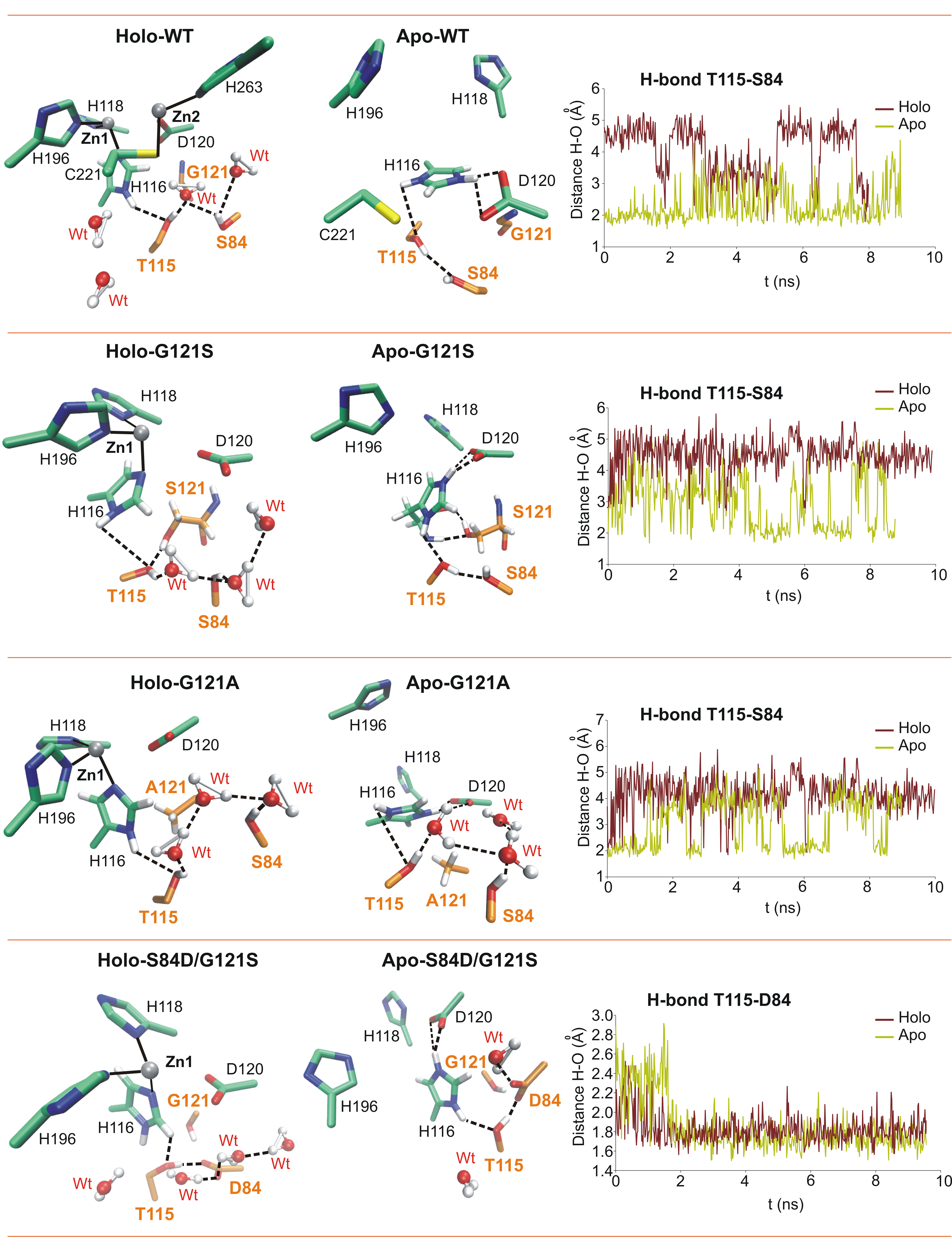

Supplement: Figure S3 — Structures of holo and apo derivatives of wild type (WT), G121S, G121A and S84D/G121S SPM-1 after 10 ns of simulation. Metal-ligand bonds are shown in solid lines and second-shell interactions in dashed lines. On the right, evolution of H-O distances of possible H bridges between residues 115 and 84. For simplicity, Zn2 and Zn2-ligands C221 and H263 are omitted in most representations. (TIF) [file ppat.1003817.s003.tif]
